# Supplementary material for: A PCR-free rapid protocol for one-pot construction of highly diverse genetic libraries
Source: PLoS One. 2022 Oct 31;17(10):e0276338. doi: 10.1371/journal.pone.0276338 (PMC9621413; doi:10.1371/journal.pone.0276338)
Supplement: S2 Fig — Frequency of each amino acid at each position of the sequenced amplicon, including reference sequence. Residue numbers refer to position within the complete HARPin gene. Randomized residues are depicted in red. Analysis performed on dataset containing de-duplicated, full-length sequences. (PDF) [file pone.0276338.s002.pdf]

	N-Cap									AR1														
Reference	R	K	G	D	N	L	V	N	K	P	D	X	X	G	X	T	P	L	I	W	A	S	X	X
	23	24	25	26	27	28	29	30	31	32	33	34	35	36	37	38	39	40	41	42	43	44	45	46
*	0	0	0	0	0	0	0	0	0	0	0	3	4	0	3	0	0	0	0	0	0	0	4	4
A	0	0	0	0	0	0	0	0	0	0	0	3	4	0	4	0	0	0	0	0	99	0	4	4
C	0	0	0	0	0	0	0	0	0	0	0	4	4	0	5	0	0	0	0	0	0	0	4	4
D	0	0	0	100	0	0	0	0	0	0	99	3	3	0	3	0	0	0	0	0	0	0	3	3
E	0	0	0	0	0	0	0	0	0	0	0	2	2	0	2	0	0	0	0	0	0	0	2	2
F	0	0	0	0	0	0	0	0	0	0	0	6	6	0	6	0	0	0	0	0	0	0	6	6
G	0	0	100	0	0	0	0	0	0	0	0	4	5	99	5	0	0	0	0	0	0	0	5	5
H	0	0	0	0	0	0	0	0	0	0	0	3	3	0	4	0	0	0	0	0	0	0	3	3
I	0	0	0	0	0	0	0	0	0	0	0	6	5	0	5	0	0	0	99	0	0	0	5	4
K	0	100	0	0	0	0	0	0	99	0	0	3	3	0	3	0	0	0	0	0	0	0	3	3
L	0	0	0	0	0	100	0	0	0	0	0	10	11	0	10	0	0	99	0	0	0	0	10	10
M	0	0	0	0	0	0	0	0	0	0	0	4	4	0	3	0	0	0	0	0	0	0	4	4
N	0	0	0	0	100	0	0	100	0	0	0	5	5	0	5	0	0	0	0	0	0	0	4	4
P	0	0	0	0	0	0	0	0	0	99	0	3	3	0	3	0	99	0	0	0	0	0	3	4
Q	0	0	0	0	0	0	0	0	0	0	0	2	2	0	2	0	0	0	0	0	0	0	2	2
R	100	0	0	0	0	0	0	0	0	0	0	7	7	0	7	0	0	0	0	0	0	0	7	7
S	0	0	0	0	0	0	0	0	0	0	0	10	9	0	9	0	0	0	0	0	0	99	9	10
T	0	0	0	0	0	0	0	0	0	0	0	5	5	0	5	100	0	0	0	0	0	0	5	5
V	0	0	0	0	0	0	100	0	0	0	0	7	7	0	7	0	0	0	0	0	0	0	8	7
W	0	0	0	0	0	0	0	0	0	0	0	3	3	0	3	0	0	0	0	99	0	0	3	3
Y	0	0	0	0	0	0	0	0	0	0	0	5	5	0	6	0	0	0	0	0	0	0	5	5

	AR1																		AR2				
Reference	G	E	I	E	T	V	R	F	L	L	E	W	G	A	D	P	H	I	L	A	X	X	R
	47	48	49	50	51	52	53	54	55	56	57	58	59	60	61	62	63	64	65	66	67	68	69
*	0	0	0	0	0	0	0	0	0	0	0	0	0	0	0	0	0	0	0	0	4	4	0
A	0	0	0	0	0	0	0	0	0	0	0	0	0	99	0	0	0	0	0	99	4	4	0
C	0	0	0	0	0	0	0	0	0	0	0	0	0	0	0	0	0	0	0	0	4	4	0
D	0	0	0	0	0	0	0	0	0	0	0	0	0	0	99	0	0	0	0	0	4	3	0
E	0	99	0	99	0	0	0	0	0	0	99	0	0	0	0	0	0	0	0	0	2	2	0
F	0	0	0	0	0	0	0	99	0	0	0	0	0	0	0	0	0	0	1	0	6	7	0
G	99	0	0	0	0	0	0	0	0	0	0	0	99	0	0	0	0	0	0	1	5	5	0
H	0	0	0	0	0	0	0	0	0	0	0	0	0	0	0	0	99	0	0	0	3	3	0
I	0	0	99	0	0	0	0	0	0	0	0	0	0	0	0	0	0	99	0	0	5	5	0
K	0	0	0	0	0	0	0	0	0	0	0	0	0	0	0	0	0	0	0	0	3	3	0
L	0	0	0	0	0	0	0	1	99	99	0	0	0	0	0	0	0	0	99	0	10	11	0
M	0	0	0	0	0	0	0	0	0	0	0	0	0	0	0	0	0	0	0	0	3	4	0
N	0	0	0	0	0	0	0	0	0	0	0	0	0	0	0	0	0	0	0	0	4	4	0
P	0	0	0	0	0	0	0	0	0	0	0	0	0	0	0	99	0	0	0	0	3	3	0
Q	0	0	0	0	0	0	0	0	0	0	0	0	0	0	0	0	0	0	0	0	2	2	0
R	0	0	0	0	0	0	99	0	0	0	0	0	0	1	0	0	0	0	0	0	7	6	99
S	0	0	0	0	0	0	0	0	0	0	0	0	0	0	0	0	0	0	0	0	10	10	0
T	0	0	0	0	99	0	0	0	0	0	0	0	0	0	0	0	0	0	0	0	5	5	0
V	0	0	0	0	0	99	0	0	0	0	0	0	0	0	0	0	0	0	0	0	7	7	0
W	0	0	0	0	0	0	0	0	0	0	0	99	0	0	0	0	0	0	0	0	3	3	0
Y	0	0	0	0	0	0	0	0	0	0	0	0	0	0	0	0	0	0	0	0	6	5	0

	AR2																						
Reference	X	S	A	L	S	L	A	S	X	X	G	Y	T	D	I	V	G	L	L	L	E	R	D
	70	71	72	73	74	75	76	77	78	79	80	81	82	83	84	85	86	87	88	89	90	91	92
*	4	0	0	0	0	0	0	0	3	4	0	0	0	0	0	0	0	0	0	0	1	0	1
A	4	0	99	0	0	0	99	0	4	5	0	0	0	0	0	0	0	0	1	1	0	1	0
C	5	0	1	0	0	0	1	0	5	4	0	0	0	0	0	0	0	0	0	0	0	0	0
D	3	0	0	0	0	0	0	0	3	3	0	0	0	99	0	0	0	0	0	0	0	0	99
E	2	0	0	0	0	0	0	0	2	2	0	0	0	0	0	0	0	0	0	0	99	0	0
F	6	0	0	0	0	0	0	0	6	6	0	0	0	0	0	0	0	0	0	0	0	0	0
G	5	0	0	0	0	0	0	0	5	5	99	0	0	0	0	0	99	0	0	0	0	0	0
H	3	0	0	0	0	0	0	0	3	3	0	0	0	0	1	0	0	0	0	0	0	0	0
I	5	0	0	0	0	0	0	1	5	4	0	1	0	0	99	0	0	0	0	0	0	0	0
K	3	0	0	0	0	0	0	0	3	2	0	0	0	0	0	0	0	0	0	0	0	0	0
L	11	0	0	99	0	99	0	0	10	11	0	0	0	0	0	0	0	99	99	99	0	0	0
M	3	0	0	0	0	0	0	0	3	3	0	0	0	0	0	0	0	0	0	0	0	0	0
N	3	0	0	0	0	0	0	0	4	3	0	0	0	0	0	0	0	0	0	0	0	0	0
P	3	0	0	1	0	0	0	0	4	4	0	0	0	0	0	0	0	0	0	0	0	0	0
Q	2	0	0	0	0	0	0	0	2	3	0	0	0	0	0	0	0	0	0	0	0	0	0
R	7	0	0	0	0	0	0	0	7	7	0	0	0	1	0	1	1	0	0	0	0	99	0
S	9	99	0	0	99	1	0	99	10	10	0	0	0	0	0	0	0	1	0	0	0	0	0
T	4	0	0	0	0	0	0	0	5	4	0	0	99	0	0	0	0	0	0	0	0	0	0
V	8	0	0	0	1	0	0	0	7	7	0	0	0	0	0	99	0	0	0	0	0	0	0
W	3	0	0	0	0	0	0	0	3	3	0	0	0	0	0	0	0	0	0	0	0	0	0
Y	5	0	0	0	0	0	0	0	5	5	0	99	1	0	0	0	0	0	0	0	0	0	0

	AR2					AR3																		
Reference	V	D	I	N	I	Y	D	X	X	G	X	T	P	L	L	Y	A	V	X	X	N	H	V	K
	93	94	95	96	97	98	99	100	101	102	103	104	105	106	107	108	109	110	111	112	113	114	115	116
*	0	1	0	0	0	0	0	3	3	0	3	0	0	0	0	0	0	0	3	3	0	0	0	0
A	0	0	0	0	0	0	0	3	3	0	3	0	1	0	0	0	99	0	4	4	0	0	0	0
C	0	0	0	0	0	0	0	4	4	0	4	0	0	0	0	0	1	1	4	5	0	0	0	0
D	0	99	0	0	0	0	99	3	3	0	3	0	0	0	0	0	0	0	3	3	0	0	0	0
E	0	0	0	0	0	0	0	1	2	0	1	0	0	0	0	0	0	0	2	2	0	0	0	0
F	0	0	0	0	0	0	0	8	9	0	9	0	0	1	0	0	0	0	8	9	0	0	0	0
G	0	0	0	0	0	0	0	3	3	99	3	0	0	0	0	0	0	0	5	5	0	0	0	0
H	0	0	1	0	1	0	0	4	4	0	4	0	0	0	0	0	0	0	3	2	0	98	0	0
I	0	0	99	0	99	0	0	7	6	0	7	0	0	0	0	1	0	0	5	6	0	0	0	0
K	0	0	0	0	0	0	0	2	3	0	2	0	0	0	0	0	0	0	2	2	0	0	0	99
L	0	0	0	0	0	1	0	11	11	0	11	0	0	98	98	0	0	0	11	10	0	0	0	0
M	0	0	0	0	0	0	0	3	3	0	3	0	0	0	0	0	0	0	3	3	0	0	0	0
N	0	0	0	99	0	0	0	5	5	0	5	0	0	0	0	0	0	0	4	4	98	0	0	0
P	0	0	0	0	0	0	0	4	4	0	4	0	99	0	0	0	0	0	4	3	0	1	0	0
Q	0	0	0	1	0	0	0	2	2	0	2	0	0	0	0	0	0	0	2	1	0	0	0	0
R	1	0	0	0	0	0	1	5	5	0	5	0	0	0	0	0	0	0	6	5	0	0	1	0
S	0	0	0	0	0	0	0	11	10	0	10	0	0	0	0	0	0	0	10	11	0	0	0	0
T	0	0	0	0	0	0	0	6	5	0	5	99	0	0	0	0	0	0	5	5	0	0	0	0
V	99	0	0	0	0	0	0	5	6	0	6	0	0	0	1	0	0	99	7	8	0	0	99	0
W	0	0	0	0	0	0	0	2	2	0	2	0	0	0	0	0	0	0	3	3	0	0	0	0
Y	0	0	0	0	0	99	0	7	6	0	6	0	0	0	0	98	0	0	6	5	0	0	0	0

	AR3														C-Cap			
Reference	C	V	E	A	L	L	A	R	G	A	D	L	T	T	E	A	X	X
	117	118	119	120	121	122	123	124	125	126	127	128	129	130	131	132	133	134
*	0	0	0	0	0	0	0	0	0	0	0	0	0	0	0	0	3	3
A	0	0	0	99	0	0	99	0	0	99	0	0	0	0	0	99	3	4
C	99	1	0	0	0	0	0	0	0	0	0	0	0	0	0	0	4	4
D	0	0	0	0	0	0	0	0	0	0	99	0	0	0	0	0	3	3
E	0	0	98	0	0	0	0	0	0	0	0	0	0	0	99	0	2	2
F	0	0	0	0	0	0	0	0	0	0	0	0	0	0	0	0	7	7
G	0	0	0	1	0	0	0	0	99	0	0	0	0	0	0	0	4	4
H	0	0	0	0	0	0	0	0	0	0	0	0	0	0	0	0	3	4
I	0	0	0	0	0	0	0	0	0	0	0	0	0	0	0	0	6	6
K	0	0	0	0	0	0	0	0	0	0	0	0	0	0	0	0	3	3
L	0	0	0	0	99	99	0	0	0	0	0	99	0	0	0	0	10	10
M	0	0	0	0	0	0	0	0	0	0	0	0	0	0	0	0	4	3
N	0	0	0	0	0	0	0	0	0	0	0	0	0	0	0	0	5	5
P	0	0	0	0	0	0	0	0	0	0	0	0	0	0	0	0	2	4
Q	0	0	0	0	0	0	0	0	0	0	0	0	0	0	0	0	2	2
R	0	0	1	0	0	0	0	99	0	0	0	0	0	0	0	0	6	6
S	0	0	0	0	0	0	0	0	0	0	0	0	0	0	0	0	10	10
T	0	0	0	0	0	0	0	0	0	0	0	0	99	99	0	0	5	6
V	1	99	0	0	1	0	0	0	0	0	0	0	0	0	0	0	7	6
W	0	0	0	0	0	0	0	0	0	0	0	0	0	0	0	0	3	2
Y	0	0	0	0	0	0	0	0	0	0	0	0	0	0	0	0	6	6

	C-Cap																
Reference	G	X	T	P	M	D	L	A	V	X	X	G	Y	R	K	V	Q
	135	136	137	138	139	140	141	142	143	144	145	146	147	148	149	150	151
*	0	3	0	0	0	0	0	0	0	3	3	0	0	0	0	0	0
A	0	4	0	0	0	0	0	100	0	4	4	0	0	0	0	0	0
C	0	4	0	0	0	0	0	0	0	4	4	0	0	0	0	0	0
D	0	4	0	0	0	99	0	0	0	3	3	0	0	0	0	0	0
E	0	2	0	0	0	0	0	0	0	2	2	0	0	0	0	0	0
F	0	7	0	0	0	0	0	0	0	6	6	0	0	0	0	0	0
G	99	4	0	0	0	0	0	0	0	4	4	100	0	0	0	0	0
H	0	4	0	0	0	0	0	0	0	4	4	0	0	0	0	0	0
I	0	6	0	0	0	0	0	0	0	7	5	0	0	0	0	0	0
K	0	3	0	0	0	0	0	0	0	3	2	0	0	0	100	0	0
L	0	10	0	0	0	0	100	0	0	10	10	0	0	0	0	0	0
M	0	3	0	0	99	0	0	0	0	4	3	0	0	0	0	0	0
N	0	5	0	0	0	0	0	0	0	5	5	0	0	0	0	0	0
P	0	4	0	100	0	0	0	0	0	4	5	0	0	0	0	0	0
Q	0	2	0	0	0	0	0	0	0	2	2	0	0	0	0	0	100
R	0	6	0	0	0	0	0	0	0	6	6	0	0	100	0	0	0
S	0	10	0	0	0	0	0	0	0	10	10	0	0	0	0	0	0
T	0	6	100	0	0	0	0	0	0	6	6	0	0	0	0	0	0
V	0	7	0	0	0	0	0	0	100	6	6	0	0	0	0	100	0
W	0	2	0	0	0	0	0	0	0	2	2	0	0	0	0	0	0
Y	0	6	0	0	0	0	0	0	0	5	6	0	100	0	0	0	0

Mean AA Frequency at Randomized Residues

Average	Std Dev
3.4	0.5
3.8	0.5
4.2	0.4
3.1	0.3
1.9	0.3
6.9	1.1
4.4	0.8
3.4	0.6
5.5	0.9
2.7	0.5
10.4	0.5
3.4	0.5
4.5	0.5
3.6	0.7
2.0	0.3
6.3	0.8
9.9	0.6
5.2	0.6
6.8	0.8
2.7	0.5
5.6	0.6

S2 Fig. Amino acid frequency analysis. Frequency of each amino acid at each position of the sequenced amplicon, including reference sequence. Residue numbers refer to position within the complete HARPin gene. Randomized residues are depicted in red. Analysis performed on dataset containing de-duplicated, full-length sequences.
