# Supplementary material for: A PCR-free rapid protocol for one-pot construction of highly diverse genetic libraries
Source: PLoS One. 2022 Oct 31;17(10):e0276338. doi: 10.1371/journal.pone.0276338 (PMC9621413; doi:10.1371/journal.pone.0276338)
Supplement: S1 Table — (PDF) [file pone.0276338.s003.pdf]

S1 Table. Primer Sequences

Primer	Sequence
NF1	gaaattaatacgactcactatagggagaccacaacggtttccctctagaaataattttgtttaactttaagaaggaggatatatccatgg
NF2	/5-Phos/gatccggtgattctttgagtattcatcagctggccgcacaaggggagcttgatcagctgaaggaacatttgcgtaagg
NF3	/5-Phos/gtgataatttggtaaataagccggacNNKNNKggaNNKactccgctgatttgggctagtNNKNNKggcgagatcgaaaccgtccgcttcc
NF4	/5-Phos/tgttggagtggggcgcggacccacatattttggccNNKNNKcgtNNKtctgccctgtctcttgcatctNNKNNKggatatacagacatcg
NF5	/5-Phos/tcggtctgctgcttgagcgtgacgttga
NF6	/5-Phos/catcaacatttacgatNNKNNKggtNNKacgcctttgttatatgctgtcNNKNNKaaccacgtaaagtg
NF7	/5-Phos/tgtcgaggcgttattggctcgcggtgcggat
NF8	/5-Phos/ctgaccactgaagcgNNKNNKggtNNKacacctatggaccttgctgtgNNKNNKggttaccgtaaggtg
NF9	/5-Phos/caacaagtaatcgagaatcacatccttaaattgtttcagtcaaaccttgttcctgcggacccagaaggtaccgga
CR	tttattccacgccgcccccgtccttttgccttccttgaggagagtaggacctccggtaccttctgggtccgc
SP1	Gaatcaccggatcccatggatatatcctc/3-Phos/
SP2	cttatttaccaaattatcacccttacgcaaatgttcct/3-Phos/
SP3	cccactccaacaggaagcggacg/3-Phos/
SP4	cagcagaccgacgatgtctgtatatcc/3-Phos/
SP5	atcgtaaatgttgatgtcaacgtcacgc/3-Phos/
SP6	cgcctcgacacactttacgtggtt/3-Phos/
SP7	cttcagtggtcagatccgcaccg/3-Phos/
SP8	ctcgattacttgttgcaccttacggtaac/3-Phos/
HARPin_F	ggagaccacaacggtttcc
HARPin_R	ttctgggtccgcagg
NGS_F	acactctttccctacacgacgctcttccgatcttgcgtaagggtgataatttgg
NGS_R	gactggagttcagacgtgtgctcttccgatctttgcaccttacggtaacc

Note: Sequences highlighted in blue represent the Illumina adapters appended to the sequenced amplicon
