# Supplementary material for: A PCR-free rapid protocol for one-pot construction of highly diverse genetic libraries
Source: PLoS One. 2022 Oct 31;17(10):e0276338. doi: 10.1371/journal.pone.0276338 (PMC9621413; doi:10.1371/journal.pone.0276338)
Supplement: S2 Table — (PDF) [file pone.0276338.s004.pdf]

S2 Table. qPCR Standard Curve Results

	Starting Quantity (copies)	C_T	Mean C_T
Std-1	$5x10^9$	4.51	4.71
		4.79	
		4.82	
Std-2	$5x10^8$	9.82	9.89
		10.11	
		9.75	
Std-3	$5x10^7$	14.15	14.24
		14.32	
		14.26	
Std-4	$5x10^7$	18.57	18.80
		18.57	
		19.26	
Std-5	$5x10^5$	23.20	23.23
		23.21	
		23.28	
