# Supplementary figures and images for: A PCR-free rapid protocol for one-pot construction of highly diverse genetic libraries
Source: PLoS One. 2022 Oct 31;17(10):e0276338. doi: 10.1371/journal.pone.0276338 (PMC9621413; doi:10.1371/journal.pone.0276338)

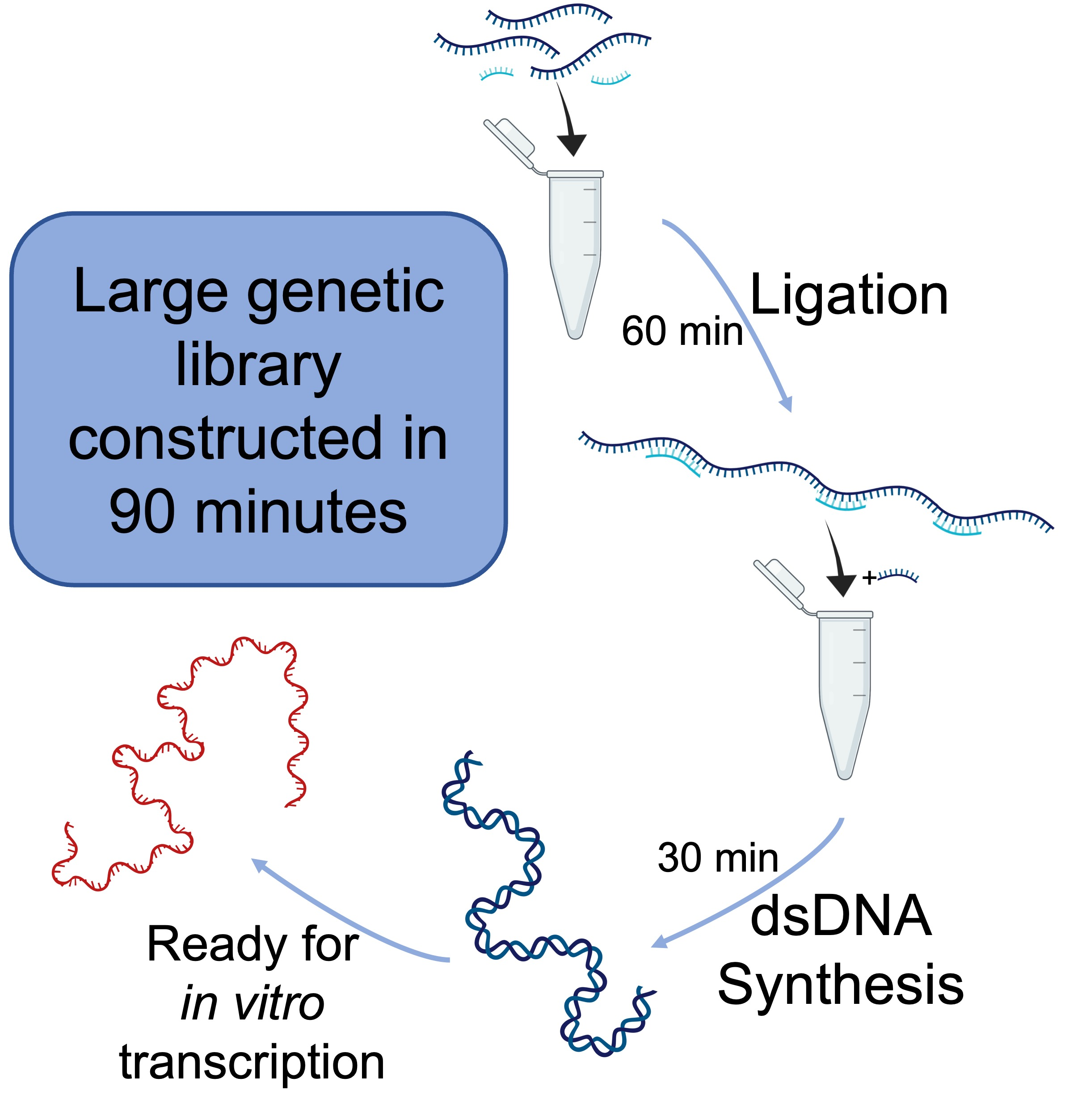

Supplement: S1 Graphical abstract — (TIF) [file pone.0276338.s005.tif]
