# Supplementary material for: A PCR-free rapid protocol for one-pot construction of highly diverse genetic libraries
Source: PLoS One. 2022 Oct 31;17(10):e0276338. doi: 10.1371/journal.pone.0276338 (PMC9621413; doi:10.1371/journal.pone.0276338)
Supplement: S1 Fig — Nucleotide frequency at each position of the sequenced amplicon, including reference sequence. Nucleotide numbers refer to position within the complete HARPin gene. Randomized codons are depicted in red. Analysis performed on dataset containing de-duplicated, full-length sequences. (PDF) [file pone.0276338.s001.pdf]

	181	182	183	184	185	186	187	188	189	190	191	192	193	194	195	196	197	198	199	200	201	202	203	204	205	206	207	208	209	210	211	212	213	214	215	216	217	218	219	220	221	222	223	224	225	226	227	228	229	230	231	232
	NF2									NF3																																										
Reference	T	G	C	G	T	A	A	G	G	G	T	G	A	T	A	A	T	T	T	G	G	T	A	A	A	T	A	A	G	C	C	G	G	A	C	N	N	K	N	N	K	G	G	A	N	N	K	A	C	T	C	C
A	0	0	0	0	0	100	100	0	0	0	0	0	100	0	100	100	0	0	0	0	0	0	100	100	100	0	100	100	0	0	0	0	0	100	0	29	28	0	28	28	0	0	0	100	28	28	0	100	0	0	0	0
T	100	0	0	0	100	0	0	0	0	0	100	0	0	100	0	0	100	100	100	0	0	100	0	0	0	100	0	0	0	0	0	0	0	0	0	32	32	58	32	32	57	0	0	0	33	32	62	0	0	100	0	0
C	0	0	100	0	0	0	0	0	0	0	0	0	0	0	0	0	0	0	0	0	0	0	0	0	0	0	0	0	0	100	100	0	0	0	100	20	18	0	19	19	0	0	0	0	19	17	0	0	100	0	100	100
G	0	100	0	100	0	0	0	100	100	100	0	100	0	0	0	0	0	0	0	100	100	0	0	0	0	0	0	0	99	0	0	100	100	0	0	20	22	42	21	21	42	100	100	0	21	23	37	0	0	0	0	0

	233	234	235	236	237	238	239	240	241	242	243	244	245	246	247	248	249	250	251	252	253	254	255	256	257	258	259	260	261	262	263	264	265	266	267	268	269	270	271	272	273	274	275	276	277	278	279	280	281	282	283	284
	NF3																																															NF4				
Reference	G	C	T	G	A	T	T	T	G	G	G	C	T	A	G	T	N	N	K	N	N	K	G	G	C	G	A	G	A	T	C	G	A	A	A	C	C	G	T	C	C	G	C	T	T	C	C	T	G	T	T	G
A	0	0	0	0	100	0	0	0	0	0	0	0	0	100	0	0	27	27	0	27	27	0	0	0	0	0	99	1	99	0	0	0	100	100	100	0	0	0	0	0	0	0	0	0	0	0	0	0	0	0	0	0
T	0	0	100	0	0	100	100	100	0	0	0	0	100	0	0	99	31	32	56	33	31	57	0	0	0	0	0	0	0	99	0	0	0	0	0	0	0	0	99	0	0	0	0	99	100	0	0	99	1	99	100	0
C	0	99	0	0	0	0	0	0	0	0	0	99	0	0	0	0	19	19	0	19	19	0	0	0	99	0	0	0	0	0	99	0	0	0	0	100	100	0	0	99	100	1	99	0	0	100	99	0	0	0	0	0
G	99	0	0	99	0	0	0	0	99	100	100	0	0	0	99	0	23	22	44	21	23	42	100	100	0	99	1	99	0	0	0	99	0	0	0	0	0	99	0	0	0	99	0	0	0	0	0	0	99	0	0	99

	285	286	287	288	289	290	291	292	293	294	295	296	297	298	299	300	301	302	303	304	305	306	307	308	309	310	311	312	313	314	315	316	317	318	319	320	321	322	323	324	325	326	327	328	329	330	331	332	333	334	335	336
	NF4																																																			
Reference	G	A	G	T	G	G	G	G	C	G	C	G	G	A	C	C	C	A	C	A	T	A	T	T	T	T	G	G	C	C	N	N	K	N	N	K	C	G	T	N	N	K	T	C	T	G	C	C	C	T	G	T
A	0	99	0	0	0	0	0	0	0	0	0	0	0	99	1	0	0	99	1	99	1	99	1	0	0	0	0	0	0	0	26	28	0	27	27	0	0	0	0	24	26	0	0	0	0	0	0	0	0	0	0	0
T	0	0	0	99	0	0	0	0	0	0	0	0	0	0	0	0	0	0	0	0	99	1	99	100	100	100	1	0	0	0	34	31	59	34	33	58	0	0	99	34	33	57	99	1	99	1	0	0	0	99	1	99
C	0	0	0	0	0	0	0	0	99	1	99	0	0	0	99	100	100	1	99	1	0	0	0	0	0	0	0	0	99	100	18	19	0	19	19	0	99	1	0	19	18	0	0	99	1	0	99	100	100	1	0	0
G	100	1	99	1	99	100	100	99	1	99	1	99	100	0	0	0	0	0	0	0	0	0	0	0	0	0	99	99	1	0	21	22	41	21	21	41	0	99	1	23	23	42	0	0	0	99	1	0	0	0	99	1

	337	338	339	340	341	342	343	344	345	346	347	348	349	350	351	352	353	354	355	356	357	358	359	360	361	362	363	364	365	366	367	368	369	370	371	372	373	374	375	376	377	378	379	380	381	382	383	384	385	386	387	388
	NF4																																	NF5																		
Reference	C	T	C	T	T	G	C	A	T	C	T	N	N	K	N	N	K	G	G	A	T	A	T	A	C	A	G	A	C	A	T	C	G	T	C	G	G	T	C	T	G	C	T	G	C	T	T	G	A	G	C	G
A	0	0	0	0	0	0	0	99	1	0	0	25	25	0	22	25	0	0	0	99	1	99	1	99	1	99	1	99	1	99	1	0	0	0	0	0	0	0	0	0	0	0	0	0	0	0	0	0	99	1	0	0
T	1	99	1	99	100	1	0	0	99	1	99	32	31	58	34	32	57	1	0	0	99	1	99	1	0	0	0	0	0	0	99	1	0	99	1	0	0	99	1	99	1	0	99	1	0	99	100	1	0	0	0	0
C	99	1	99	1	0	0	99	1	0	99	1	21	20	0	22	20	0	0	0	0	0	0	0	0	99	1	0	0	99	1	0	99	1	0	99	1	0	0	99	1	0	99	1	0	99	1	0	0	0	0	99	1
G	0	0	0	0	0	99	1	0	0	0	0	22	24	41	22	23	42	99	100	1	0	0	0	0	0	0	99	1	0	0	0	0	99	1	0	99	100	1	0	0	99	1	0	99	1	0	0	99	1	99	1	99

	389	390	391	392	393	394	395	396	397	398	399	400	401	402	403	404	405	406	407	408	409	410	411	412	413	414	415	416	417	418	419	420	421	422	423	424	425	426	427	428	429	430	431	432	433	434	435	436	437	438	439	440
	NF5									NF6																																										
Reference	T	G	A	C	G	T	T	G	A	C	A	T	C	A	A	C	A	T	T	T	A	C	G	A	T	N	N	K	N	N	K	G	G	T	N	N	K	A	C	G	C	C	T	T	T	G	T	T	A	T	A	T
A	0	0	99	1	0	0	0	0	99	1	99	1	0	99	100	1	99	1	0	0	99	1	0	99	1	27	27	0	27	27	0	0	0	0	27	27	0	99	1	0	0	0	0	0	0	0	0	0	99	1	98	1
T	99	1	0	0	0	99	100	1	0	0	0	99	1	0	0	0	0	99	100	100	1	0	0	0	99	37	35	66	36	35	65	1	0	99	36	36	68	1	0	0	0	0	99	100	99	1	99	100	1	99	1	99
C	0	0	0	99	1	0	0	0	0	99	1	0	99	1	0	99	1	0	0	0	0	0	99	1	0	20	21	0	21	20	0	0	0	0	20	19	0	0	99	1	99	100	1	0	0	0	0	0	0	0	1	0
G	1	99	1	0	99	1	0	99	1	0	0	0	0	0	0	0	0	0	0	0	0	0	99	1	0	16	17	33	16	18	34	99	100	1	18	18	32	0	0	99	1	0	0	0	0	99	1	0	0	0	0	0

	441	442	443	444	445	446	447	448	449	450	451	452	453	454	455	456	457	458	459	460	461	462	463	464	465	466	467	468	469	470	471	472	473	474	475	476	477	478	479	480	481	482	483	484	485	486	487	488	489	490	491	492
	NF6																										NF7																									
Reference	G	C	T	G	T	C	N	N	K	N	N	K	A	A	C	C	A	C	G	T	A	A	A	G	T	G	T	G	T	C	G	A	G	G	C	G	T	T	A	T	T	G	G	C	T	C	G	C	G	G	T	G
A	0	0	0	0	0	0	25	26	0	25	23	0	99	99	1	0	99	1	0	0	99	99	99	1	0	0	0	0	0	0	0	99	1	0	0	0	0	0	99	1	0	0	0	0	0	0	0	0	0	0	0	0
T	1	0	99	1	99	1	35	34	62	39	35	62	1	0	0	0	0	0	0	99	1	0	0	0	99	1	99	1	99	1	0	0	0	0	0	0	99	100	1	99	100	0	0	0	99	0	0	0	0	0	99	0
C	0	99	1	0	0	99	20	19	0	14	20	0	0	0	99	100	1	99	1	0	0	0	0	0	0	0	0	0	0	99	1	0	0	0	99	1	0	0	0	0	0	0	0	99	0	99	0	99	0	0	0	0
G	99	1	0	99	1	0	20	21	37	22	22	38	0	0	0	0	0	0	99	1	0	0	0	99	1	99	1	99	1	0	99	1	99	100	1	99	1	0	0	0	0	100	100	0	0	0	99	1	100	100	0	99

	493	494	495	496	497	498	499	500	501	502	503	504	505	506	507	508	509	510	511	512	513	514	515	516	517	518	519	520	521	522	523	524	525	526	527	528	529	530	531	532	533	534	535	536	537	538	539	540	541	542	543	544
	NF7					NF8																																														
Reference	C	G	G	A	T	C	T	G	A	C	C	A	C	T	G	A	A	G	C	G	N	N	K	N	N	K	G	G	T	N	N	K	A	C	A	C	C	T	A	T	G	G	A	C	C	T	T	G	C	T	G	T
A	0	0	0	99	0	0	0	0	100	0	0	99	0	0	0	100	99	0	0	0	29	29	0	29	28	0	0	0	0	28	27	0	100	0	100	0	0	0	100	0	0	0	100	0	0	0	0	0	0	0	0	0
T	0	0	0	0	100	0	99	0	0	0	0	0	0	100	0	0	0	0	0	0	34	34	62	32	33	66	0	0	100	33	33	66	0	0	0	0	0	100	0	100	0	0	0	0	0	100	100	0	0	100	0	100
C	99	0	0	0	0	100	0	0	0	100	100	0	100	0	0	0	0	0	99	0	17	17	0	21	20	0	0	0	0	19	20	0	0	100	0	100	100	0	0	0	0	0	0	100	100	0	0	0	100	0	0	0
G	0	100	100	0	0	0	0	99	0	0	0	0	0	0	100	0	0	100	0	100	20	20	38	19	19	34	100	100	0	20	20	33	0	0	0	0	0	0	0	0	100	100	0	0	0	0	0	100	0	0	100	0

	545	546	547	548	549	550	551	552	553	554	555	556	557	558	559	560	561	562	563	564	565	566	567	568	569
	NF8																								
Reference	G	N	N	K	N	N	K	G	G	T	T	A	C	C	G	T	A	A	G	G	T	G	C	A	A
A	0	29	27	0	27	27	0	0	0	0	0	100	0	0	0	0	100	100	0	0	0	0	0	100	100
T	0	31	33	63	32	31	62	0	0	100	100	0	0	0	0	100	0	0	0	0	100	0	0	0	0
C	0	20	20	0	21	22	0	0	0	0	0	0	100	100	0	0	0	0	0	0	0	0	100	0	0
G	100	19	20	37	20	20	38	100	100	0	0	0	0	0	100	0	0	0	100	100	0	100	0	0	0

Mean Nucleotide Frequency at Randomized Codons

N		N		K	
Average	Std Dev	Average	Std Dev	Average	Std Dev
26.8	1.9	26.9	1.3	0.0	0.0
33.7	2.1	32.9	1.5	61.1	3.8
19.4	1.7	19.3	1.2	0.0	0.0
20.3	1.9	21.0	1.9	38.4	3.7

S1 Fig. Nucleotide frequency analysis. Nucleotide frequency at each position of the sequenced amplicon, including reference sequence. Nucleotide numbers refer to position within the complete HARPin gene. Randomized codons are depicted in red. Analysis performed on dataset containing de-duplicated, full-length sequences.
