# Supplementary material for: A PCR-free rapid protocol for one-pot construction of highly diverse genetic libraries
Source: PLoS One. 2022 Oct 31;17(10):e0276338. doi: 10.1371/journal.pone.0276338 (PMC9621413; doi:10.1371/journal.pone.0276338)
Supplement: Attachment — Submitted filename: Response to Reviewers.docx [file pone.0276338.s006.docx]

**Response to Reviewers**

We thank the Reviewers for their helpful comments. Based on their concerns, we have added the following new data to the manuscript:

Figure 4. Next-Generation Sequencing Results

Figure S1. Nucleotide Frequency Grid Profile

Figure S2. Amino Acid Frequency Grid Profile

**Review Comments to the Author**

**Reviewer #1**: The manuscript entitled "A PCR-free rapid protocol for one-pot construction of highly diverse libraries for in vitro protein engineering" by Woolley and Chen Claims a fast and simple mRNA library preparation method using ligation and elongation reaction. The authors utilized T4 DNA ligase to assemble and increase the size of the fragment with the aid of splint oligos. Bst2.0 polymerase then synthesized DNA from the CR oligo and strand-displaced the residual splint oligos to complete dsDNA. Simultaneous in vitro transcription could transcribe a sizeable mRNA library without further purification.

The method is clear, and the final result (mRNA library) is promising. I believe the authors can improve the manuscript with the following suggestions.

Major
1) It is unclear the actual diversity of the library by only showing the number of the library. Can you provide the sequencing data?

*Yes. The final mRNA (Figure 1, Step 4) was reversed transcribed and subjected to deep sequencing after PCR amplification to include the adaptors. The new data is presented in* ***Figures 4, S1, and S2****. A total of 293,067 sequence reads were obtained from which 244,974 were unique. The small percentage of duplicate sequences is likely an artifact of the PCR reaction needed to append the adaptors needed for sequencing. A total of 162,442 unique and full-length reads were obtained. Among the unique sequence reads, 78,808 contain the full-length gene without any internal stop codons.*

*The following text was added to the manuscript (page 8, lines: 162-179):*

*“Next-Generation Sequencing (NGS) was used to assess the quality of this new mRNA library. Five hundred nanograms of the in vitro transcription mRNA product (from Figure 1, Step 4) was reverse transcribed and then PCR amplified to append the NGS linkers. Approximately 750 nanograms of the PCR product was subjected to the MiSeq Next-Generation Sequencing reaction and yielded a total of 293,067 sequence reads (****Figure 4A****). After removing duplicate sequences and those of incorrect length, the cleaned dataset contained 162,422 unique reads (****Figure 4A****). The frequency of each base at the randomized codon position ranges between 19.3% to 33.7% for the N nucleotides and 38.4% to 61.1% for the K nucleotides (****Figure S1****), which is consistent with oligos synthesized with machine mixing (****Figure 4B, S1****). The average frequency of each amino acid at the randomized codon positions varies from 1.9% to 10.4%, which is consistent within that afforded by the NNK codon (****Figure 4C, S2****). Overall, 51.4% of the full-length reads contain at least one internal stop codon (****Figure 4A****). This is slightly lower than that predicted for a gene with 20 randomized NNK codons (62.5% = ^1^/_32_ STOP codon frequency at each position x 20 positions, assuming an even distribution of each possible base at the N and K positions). The final dataset of full-length sequences contained 78,808 unique variants (****Figure 4A****). These results validate the use of L&E method for in vitro library creation.”*

2) Due to the base-pairing between fragments and splint oligo, it seems you cannot randomize those regions (base-pairing regions). How many splint oligo bases do pair with fragments? Do you have a specific design role?

*It is true that regions that hybridize with the split oligo cannot be randomized. Consequently, split oligos need to be designed to bind the framework region of the scaffold. In our study, we took care to ensure that the annealing regions on each side of the SP oligos range from 10 to 20 base pairs and with an annealing temperature of approximately 40 ºC.*

*The following text has been included in the manuscript (pg 4-5; lines 69-70):*

*“…iii) splint oligos bind the framework region of the binding scaffold and include no randomized codons.­­­­”*

3) Figure 1, Steps 1-3: Residual SP oligos should be displayed in the figure because those SP oligos will not be strand-displaced until the end of step 3 by Bst2.0 polymerase.

*Thank you for pointing this out. We have updated Figure 1 to show the SP oligos annealed to the ligation products.*

Minor
1) Page 3, lines 24-30: Showing one or two directed evolution examples will strengthen the instruction part.

*Thanks for this suggestion. The following references have been added to the Introduction section (page 3, line 28):*

*1.	Wang Y, Xue P, Cao M, Yu T, Lane ST, Zhao H. Directed Evolution: Methodologies and Applications. Chem Rev. 2021;121(20):12384-444. doi: 10.1021/acs.chemrev.1c00260.*

*2.	McLure RJ, Radford SE, Brockwell DJ. High-throughput directed evolution: a golden era for protein science. Trends Chem. 2022;4(5):378-91. doi: 10.1016/j.trechm.2022.02.004. PubMed PMID: WOS:000793458600005.*

2) Page 5, lines 8-88: "was clearly visible on the gel" - How can you be sure this band is the regions for T7 promoter and RBS without comparing ladders? I was expecting the ladder in the original image in the supplementary information.

*Thank you for pointing out this detail. Unfortunately, due to the large size of the sense-strand ssDNA ligation product (i.e., 641 nt), we were unable to identify a commercially available ssDNA ladder of equivalent size. We have previously attempted using a dsDNA ladder, but we found that there was no correlation between the dsDNA band sizes and the expected size of the ssDNA fragments (e.g., the largest band in the Group 1 ligation 1 (Fig 1A) is expected to be 258 nt but ran at the same rate at the 400 bp ladder band).*

*We posit that the transcription of a single mRNA product of the correct size should indirectly confirm the synthesis of the correct sense-strand ssDNA harboring both the primer and the coding sequence.*

*All the gel images presented in the manuscript contain all the visible bands on the gel. Uncropped original gel images are provided in the supporting information S1_raw_images file.*

*The manuscript text is modified as follows (page 6, line 98-100)*

*“The largest product is presumed to correspond to the ligation product of all the sense oligos (620 nt), which includes the regions for the T7 promoter and ribosome binding site (RBS) (****Figure 2A****, green arrow).”*

3) Page 7, lines 138-146: Although this manuscript focuses on the methodology but adding short comments on a potential ongoing protein engineering project in the conclusion part is highly recommended.

*We are currently working on integrating this methodology with a new protein display technology developed in our lab for in vitro protein evolution. However, we feel it is premature to discuss these ongoing projects in this manuscript and prefer to mention these works in independent manuscripts in the future.*

4) Page 8, line 151: Table 1 should be Table 2. Recommend using the same table styles for Table 1 and Table 2.

*Thanks for the suggestion. We have updated the manuscript accordingly.*

**Reviewer #2**: The authors describe a ‘one-pot ligation and elongation’ method to accelerate the preparation of double-stranded DNA libraries for mRNA display of protein variants. mRNA display is a powerful technique, and the focus on library preparation should be of interest to the practitioner. That said, I do have reservations about publication of the work in current form (with the caveat that methods for DNA assembly and mRNA display are not my research focus). These fall into two categories:

1) Is the DNA assembly aspect of the work adequately placed in context? Some mention of the use of ‘splinted’ ligations in other situations may be helpful, for example:

Chen H, Weng J, Jiang K, Bao J (1990) A new method for the synthesis of a structural gene. Nucleic Acids Res. 18(4): 871-878.
-perhaps the original reference for assembly of ssDNA by enzymatic ligation using bridging oligos

Sui Z, An R, Komiyama M, Liang X (2021) Stepwise strategy for one-pot synthesis of single-stranded DNA rings from multiple short fragments. ChemBioChem. 22:1005-1011 -a recent application of one-pot, splinted ligation of ssDNA oligos to the preparation of circular ssDNA

*Thank you for this suggestion. We feel that, although ligation-based methods have been used to generate coding DNA, no one has used it in combination with DNA polymerase for creating a large synthetic library suitable for in vitro evolution. We have added the following text to help contextualize our work (pg 4; lines 59-61):*

*“We used a ligation-mediated strategy similar to that described previously [19, 20], which utilizes short antisense splint oligos that anneal at the junctions of adjacent forward strand fragments to generate the full-length gene.”*

2) Are sufficient data provided to establish the method as a sound one for mRNA display of protein libraries? Sequencing a subset of assembly products could provide an important quality control check for library construction. For example, the authors should sequence at least 20 library constructs to show the absence of insertions/deletions/mutations etc. at ligation points (i.e., that the construct remains in frame). Even better would be to use NGS (for example, do the amino acid frequencies of randomized positions match the expected frequencies, based on the library design?). Using an assembly product in translation would also be of interest, though is perhaps beyond the scope of this work.

*We appreciate the reviewer’s thoughtful point on this matter. Five-hundred ng out of 217 µg of the final transcribed mRNA (Figure 1, step 4) was reverse transcribed and sequenced after PCR amplification. A total of 244,974 unique sequence reads were obtained. A new figure 4 and the following text was added to the manuscript (page 8, lines: 162-179):*

*“Next-Generation Sequencing (NGS) was used to assess the quality of this new mRNA library. Five hundred nanograms of the in vitro transcription mRNA product (from Figure 1, Step 4) was reverse transcribed and then PCR amplified to append the NGS linkers. Approximately 750 nanograms of the PCR product was subjected to the MiSeq Next-Generation Sequencing reaction and yielded a total of 293,067 sequence reads (****Figure 4A****). After removing duplicate sequences and those of incorrect length, the cleaned dataset contained 162,422 unique reads (****Figure 4A****). The frequency of each base at the randomized codon position ranges between 19.3% to 33.7% for the N nucleotides and 38.4% to 61.1% for the K nucleotides (****Figure S1****), which is consistent with oligos synthesized with machine mixing (****Figure 4B, S1****). The average frequency of each amino acid at the randomized codon positions varies from 1.9% to 10.4%, which is consistent within that afforded by the NNK codon (****Figure 4C, S2****). Overall, 51.4% of the full-length reads contain at least one internal stop codon (****Figure 4A****). This is slightly lower than that predicted for a gene with 20 randomized NNK codons (62.5% = ^1^/_32_ STOP codon frequency at each position x 20 positions, assuming an even distribution of each possible base at the N and K positions). The final dataset of full-length sequences contained 78,808 unique variants (****Figure 4A****). These results validate the use of L&E method for in vitro library creation.”*

Minor points:
-consider adding splint oligos to the Graphical Abstract, with unique coloring -throughout, please define abbreviations (e.g., NF, CR, etc.)

*Thank you for this suggestion. We have updated the figure with different colors for the SP oligos and added definitions for the abbreviations to the caption of Figure 1 (page 5, lines 88-89):*

*“NF: N-terminal forward, SP: Splint, CR: C-terminal reverse.”*

-in the discussion, the authors may comment briefly on the specific steps involved to interface their library with mRNA display

*We have updated the discussion to comment on how our library could be used for mRNA display (page 6, lines 117-121):*

*“**Thus, transcribed mRNA can be purified using a commercial RNA clean-up kit (e.g., Zymo Research EZNA RNA Clean & Concentrate kit) and be eluted in a buffer appropriate for the subsequent in vitro protein synthesis reaction.* *Alternatively, the DNA product from Step 3 can be used directly in a coupled transcription and translation reaction for in vitro protein synthesis.”*

-Fig 2b: The authors may clarify why a ‘smear’ is obtained during dsDNA synthesis, rather than a series of resolved co-products

*The smear is likely caused by amplification of incomplete ligation products or unligated oligos that have annealed nonspecifically. The wide array of products that could potentially be formed could lead to a smear on the gel rather than distinct bands. We included the following text in the manuscript (page 6, lines 107-111):*

*“This is unsurprising given that the product of Step 2 contained multiple DNA fragments as well as excess NF oligos that can anneal to each other to yield an array of dsDNA of varying size. Five microliters of the reaction product of Step 3 was used directly for mRNA transcription in Step 4 and routinely yielded 108 – 217 µg mRNA in 20 µL reaction volume.”*

-in Methods section, line 151: ‘Table 1’ should be ‘Table 2’

*Thank you for pointing this out. The mistake has been corrected.*

**Reviewer #3**: The authors report a one-pot method for the preparation of libraries starting from oligos pools.

For minor revisions, the authors should comment on the following:
- If full-length ligation yield is between ~5-12%, how is this expected to change as the number or length of sense oligos changes for different protein targets?

*We posit that the overall ligation efficiency is inversely proportional to the number of ligation events. Consequently, increasing the length, and reducing the number, of sense oligos should yield higher ligation efficiency.*

*The following text has been added to the manuscript: (page 7, line 138-141):*

*“**Since the overall ligation efficiency is inversely proportional to the number of ligation events, increasing the length – thus reducing the number – of sense oligos may lead to a higher overall ligation efficiency.”*

- The manuscript does not provide considerations beyond transcription, which raises some questions about utility:
-- While the method may be scalable, what are the scales tentatively required for protein engineering applications?

*We thank the reviewer for this insightful question. We want to point out that purified mRNA is used as the input for mRNA display. In this work, we have demonstrated the synthesis of highly pure (Figure 2C) and concentrated (217 µg or ~1 nmol in 20 µL reaction) mRNA product using our method. This amount of mRNA is the typical starting amount used in mRNA display (Barendt, et al., ACS Comb. Set 2013, 15, 77-81. dx.doi.org/10.1021/co300135rl).*

*The following text has been added to the manuscript to highlight this point (page 6, lines 117-121):*

*“Thus, transcribed mRNA can be purified using a commercial RNA clean-up kit (e.g., Zymo Research EZNA RNA Clean & Concentrate kit) and be eluted in a buffer appropriate for the subsequent in vitro protein synthesis reaction.* *Alternatively, the DNA product from Step 3 can be used directly in a coupled transcription and translation reaction for in vitro protein synthesis.”*

-- Is the fill-length mRNA yield negatively affected due to the lack of cleanup to alternate constructs? If so, to what extent? Figure 3c demonstrates significant quantity of 300-500bp and 700+bp products. The gel is cut off below ~100bp, and smaller truncated products are not shown to the reader.

*We believe that the mRNA yield is not affected by the lack of cleanup of alternate constructs since only the full-length dsDNA contains the anti-sense of the T7 promoter. The uncropped gel image is included in the supporting information S1_raw_images file and shows that each lane (sample, negative control, positive control) had only a single band.*

-- is the one pot method compatible with in vitro translation? Or does the mRNA require cleanup/purification before translation?

*In the current work, 5 µL of the one-pot product (Step 3) was used in 20 µL of in vitro transcription reaction using the TranscriptAid T7 High Yield Transcription kit (Thermo Fisher). This reaction typically yields 100-200 µg of mRNA. Due to the difference in the buffer condition, for optimum translation efficiency, the in vitro transcribed mRNA should be cleaned up before translation. This is similar to any translation experiment in which the transcribed mRNA needs to be cleaned up using a commercial RNA Clean & Concentrator kit. Since the Nanodrop readings of the samples after cleanup showed A260/A280 ratios of approximately 2.0, we are confident that the RNA is of acceptable purity to be used directly for mRNA display or in vitro translation. In addition, since only a single mRNA product is produced, we believe no gel purification step is necessary and a simple column purification step using a kit such as RNA Clean & Concentrator -25 kit (Zymo Research) should be sufficient.*

-- For true utility with protein engineering, the compatibility with cell-free protein synthesis should be discussed or demonstrated.

*Thank you for raising this point. The following text has been added to the manuscript (page 6, lines 117-121):*

*“Thus, transcribed mRNA can be purified using a commercial RNA clean-up kit (e.g., Zymo Research EZNA RNA Clean & Concentrate kit) and be eluted in a buffer appropriate for the subsequent in vitro protein synthesis reaction.* *Alternatively, the DNA product from Step 3 can be used directly in a coupled transcription and translation reaction for in vitro protein synthesis.”*

-- is the scale used in this manuscript compatible with producing enough protein for analysis/characterization/selection?

*Yes, our method – even at the current scale – produces enough mRNA (~1 nmole in 20 µL reaction) sufficient for conventional mRNA display-based panning experiment (ref Barendt, et al., ACS Comb. Set 2013, 15, 77-81. dx.doi.org/10.1021/co300135rl).*
