# Supplementary material for: A PCR-free rapid protocol for one-pot construction of highly diverse genetic libraries
Source: PLoS One. 2022 Oct 31;17(10):e0276338. doi: 10.1371/journal.pone.0276338 (PMC9621413; doi:10.1371/journal.pone.0276338)
Supplement: Attachment — Submitted filename: Reponse to Reviewers.docx [file pone.0276338.s007.docx]

**Comments to the Author**

1. If the authors have adequately addressed your comments raised in a previous round of review and you feel that this manuscript is now acceptable for publication, you may indicate that here to bypass the “Comments to the Author” section, enter your conflict-of-interest statement in the “Confidential to Editor” section, and submit your "Accept" recommendation.

Reviewer #1: All comments have been addressed

Reviewer #2: (No Response)

Reviewer #3: (No Response)

2. Is the manuscript technically sound, and do the data support the conclusions?

The manuscript must describe a technically sound piece of scientific research with data that supports the conclusions. Experiments must have been conducted rigorously, with appropriate controls, replication, and sample sizes. The conclusions must be drawn appropriately based on the data presented.

Reviewer #1: Yes

Reviewer #2: Yes

Reviewer #3: Partly

3. Has the statistical analysis been performed appropriately and rigorously?

Reviewer #1: N/A

Reviewer #2: N/A

Reviewer #3: Yes

4. Have the authors made all data underlying the findings in their manuscript fully available?

The [PLOS Data policy](https://urldefense.com/v3/__http:/www.plosone.org/static/policies.action*sharing__;Iw!!KwNVnqRv!CP1jHEynkMEsQ8wPauQVZ2SUeWNCagPFk3teYBe67mQpJA8DSYmO6EV1fvj59GdU1bnQdfgoVH_GmYUR$) requires authors to make all data underlying the findings described in their manuscript fully available without restriction, with rare exception (please refer to the Data Availability Statement in the manuscript PDF file). The data should be provided as part of the manuscript or its supporting information, or deposited to a public repository. For example, in addition to summary statistics, the data points behind means, medians and variance measures should be available. If there are restrictions on publicly sharing data—e.g. participant privacy or use of data from a third party—those must be specified.

Reviewer #1: Yes

Reviewer #2: Yes

Reviewer #3: Yes

5. Is the manuscript presented in an intelligible fashion and written in standard English?

PLOS ONE does not copyedit accepted manuscripts, so the language in submitted articles must be clear, correct, and unambiguous. Any typographical or grammatical errors should be corrected at revision, so please note any specific errors here.

Reviewer #1: Yes

Reviewer #2: Yes

Reviewer #3: Yes

6. Review Comments to the Author

Please use the space provided to explain your answers to the questions above. You may also include additional comments for the author, including concerns about dual publication, research ethics, or publication ethics. (Please upload your review as an attachment if it exceeds 20,000 characters)

Reviewer #1: The authors carefully addressed the reviewer's comment, and the revised manuscript has improved. The manuscript is recommended for publication.

*Thank you.*

Reviewer #2: This revision satisfactorily addresses all points raised during review.

One minor comment, for the authors’ consideration: In the new paragraph describing NGS results, consider stating concisely the reasoning behind how the NGS results validate the L&E method for library creation (e.g., with respect to both assembly product integrity, and library diversity).

*We appreciate the reviewer’s suggestion. We have added the following to the NGS paragraph (page 9, lines: 178 - 180):*

“The abundance of unique full-length sequences combined with a lack of significant codon bias validate the use of L&E method for *in vitro* library creation.”

Reviewer #3: Authors should consider modifying the article title to “PCR-free rapid protocol for one-pot construction of highly diverse mRNA libraries”, as this change would address most of my concerns.

- Since the mRNA would require cleanup prior to in vitro translation, the protocol for protein engineering is no longer “one-pot”.
- It’s true that 217 ug or ~1nmol mRNA in 20 µL reaction may be useful for mRNA display, the corresponding in vitro translation reaction is unlikely to produce enough protein for analysis or characterization, and only sufficient for selection in limited cases.
Therefore, the authors should demonstrate protein production and engineering efforts if that part of their claims.
Plugging the HARPin Library into NEB’s PURExpress kit could be a good starting point. Though this would also be inconsistent with the “one-pot” claim. Such an approach could be superior to purifying the mRNA prior to in vitro translation since handling of mRNA could cause problems and variability in different hands.

*Based on the reviewer’s suggestion, we modified the title of the manuscript as follows:*

*“*A PCR-free rapid protocol for one-pot construction of highly diverse genetic libraries for in vitro protein engineering*”*

*We agree that the clean up after* in vitro *transcription would be inconsistent with the claim of a “one-pot” method. In this paper, we intend to develop the L&E method for creating highly diverse DNA/mRNA libraries for in vitro protein engineering that could bypass the conventional PCR and ligation steps. To clarify this point, we have made the following changes to the manuscript:*

***Page 6, lines 110 - 113****: “*To demonstrate the utility of the DNA library without the need of purification, five microliters of the reaction product of Step 3 was used directly for mRNA transcription (Step 4). This *in vitro* transcription reaction routinely yielded 108 – 217 µg mRNA in 20 µL reaction volume.*”*

***Page 8, lines 161 - 162****: “*We employed Next-Generation Sequencing (NGS) to assess the quality of this new library. To simulate the conditions of the library’s use in protein engineering, 500 nanograms of the *in vitro* transcription mRNA product…”

7. PLOS authors have the option to publish the peer review history of their article ([what does this mean?](https://urldefense.com/v3/__https:/journals.plos.org/plosone/s/editorial-and-peer-review-process*loc-peer-review-history__;Iw!!KwNVnqRv!CP1jHEynkMEsQ8wPauQVZ2SUeWNCagPFk3teYBe67mQpJA8DSYmO6EV1fvj59GdU1bnQdfgoVBB7Ew0c$)). If published, this will include your full peer review and any attached files.


If you choose “no”, your identity will remain anonymous, but your review may still be made public.


**Do you want your identity to be public for this peer review?** For information about this choice, including consent withdrawal, please see our [Privacy Policy](https://urldefense.com/v3/__https:/www.plos.org/privacy-policy__;!!KwNVnqRv!CP1jHEynkMEsQ8wPauQVZ2SUeWNCagPFk3teYBe67mQpJA8DSYmO6EV1fvj59GdU1bnQdfgoVMMZlCJX$).

Reviewer #1: No

Reviewer #2: **Yes:** Zachary P Gates

Reviewer #3: No
