# Supplementary material for: A PCR-free rapid protocol for one-pot construction of highly diverse genetic libraries
Source: PLoS One. 2022 Oct 31;17(10):e0276338. doi: 10.1371/journal.pone.0276338 (PMC9621413; doi:10.1371/journal.pone.0276338)
Supplement: Attachment — Submitted filename: Reponse to Reviewers.docx [file pone.0276338.s008.docx]

**Comments to the Author**

1. If the authors have adequately addressed your comments raised in a previous round of review and you feel that this manuscript is now acceptable for publication, you may indicate that here to bypass the “Comments to the Author” section, enter your conflict of interest statement in the “Confidential to Editor” section, and submit your "Accept" recommendation.

Reviewer #1: All comments have been addressed

Reviewer #2: All comments have been addressed

Reviewer #3: (No Response)

2. Is the manuscript technically sound, and do the data support the conclusions?

The manuscript must describe a technically sound piece of scientific research with data that supports the conclusions. Experiments must have been conducted rigorously, with appropriate controls, replication, and sample sizes. The conclusions must be drawn appropriately based on the data presented.

Reviewer #1: Yes

Reviewer #2: Yes

Reviewer #3: (No Response)

3. Has the statistical analysis been performed appropriately and rigorously?

Reviewer #1: Yes

Reviewer #2: N/A

Reviewer #3: (No Response)

4. Have the authors made all data underlying the findings in their manuscript fully available?

The [PLOS Data policy](https://urldefense.com/v3/__http:/www.plosone.org/static/policies.action*sharing__;Iw!!KwNVnqRv!FGnIEZKUAWwhokJWvBUKooT82xNBOaZiFuWiT6nqDA3sztpESoHvW_qjOTJHNjD5iMrd3_QbrN-sSn0m$) requires authors to make all data underlying the findings described in their manuscript fully available without restriction, with rare exception (please refer to the Data Availability Statement in the manuscript PDF file). The data should be provided as part of the manuscript or its supporting information, or deposited to a public repository. For example, in addition to summary statistics, the data points behind means, medians and variance measures should be available. If there are restrictions on publicly sharing data—e.g. participant privacy or use of data from a third party—those must be specified.

Reviewer #1: Yes

Reviewer #2: Yes

Reviewer #3: (No Response)

5. Is the manuscript presented in an intelligible fashion and written in standard English?

PLOS ONE does not copyedit accepted manuscripts, so the language in submitted articles must be clear, correct, and unambiguous. Any typographical or grammatical errors should be corrected at revision, so please note any specific errors here.

Reviewer #1: Yes

Reviewer #2: Yes

Reviewer #3: (No Response)

6. Review Comments to the Author

Please use the space provided to explain your answers to the questions above. You may also include additional comments for the author, including concerns about dual publication, research ethics, or publication ethics. (Please upload your review as an attachment if it exceeds 20,000 characters)

Reviewer #1: (No Response)

Reviewer #2: This revision satisfactorily addresses all points raised during review, and is recommended for publication.

Reviewer #3: Authors state that:

"Based on the reviewer’s suggestion, we modified the title of the manuscript as follows":
'A PCR-free rapid protocol for one-pot construction of highly diverse genetic libraries for in vitro
protein engineering' ".

This title appears to be unchanged and does not resolve my concerns.
To reiterate, the authors have not demonstrated that their method can be used for in vitro protein engineering, or that protein engineering can be done in "one-pot".

Premise of protein engineering should be removed from the title, possible options:
'A PCR-free rapid protocol for one-pot construction of highly diverse genetic libraries'
'A PCR-free rapid protocol for one-pot construction of highly diverse mRNA libraries'

*We have updated the title of the manuscript to “**A PCR-free rapid protocol for one-pot construction of highly diverse genetic libraries.”*

7. PLOS authors have the option to publish the peer review history of their article ([what does this mean?](https://urldefense.com/v3/__https:/journals.plos.org/plosone/s/editorial-and-peer-review-process*loc-peer-review-history__;Iw!!KwNVnqRv!FGnIEZKUAWwhokJWvBUKooT82xNBOaZiFuWiT6nqDA3sztpESoHvW_qjOTJHNjD5iMrd3_QbrEpCx3wY$)). If published, this will include your full peer review and any attached files.


If you choose “no”, your identity will remain anonymous but your review may still be made public.


**Do you want your identity to be public for this peer review?** For information about this choice, including consent withdrawal, please see our [Privacy Policy](https://urldefense.com/v3/__https:/www.plos.org/privacy-policy__;!!KwNVnqRv!FGnIEZKUAWwhokJWvBUKooT82xNBOaZiFuWiT6nqDA3sztpESoHvW_qjOTJHNjD5iMrd3_QbrHPqMR5O$).

Reviewer #1: No

Reviewer #2: **Yes:** Zachary P Gates

Reviewer #3: No
